# A low psoas muscle volume is associated with a poor prognosis in penile cancer

**DOI:** 10.18632/oncotarget.27719

**Published:** 2020-09-22

**Authors:** Daiji Takkamoto, Takashi Kawahara, Takashi Tokita, Jun Kasuga, Yasushi Yumura, Hiroji Uemura

**Affiliations:** ^1^Department of Urology and Renal Transportation, Yokohama City University Medical Center, Yokohama, Japan; ^2^Department of Urology, Toshiba Rinkan Hospital, Sagamihara, Japan

**Keywords:** sarcopenia, penile cancer, psoas muscle, psoas muscle volume, penectomy

## Abstract

Background: Sarcopenia was initially recognized as a marker representing the nutritional condition or aging. Recently, sarcopenia has been associated with a poor prognosis and postoperative complications. We examined the importance of sarcopenia as a predictive marker of the prognosis in penile cancer.

Materials and Methods: A total of 25 patients diagnosed with penile cancer who underwent penile resection from 2000 to 2010 were analyzed in this study. The psoas muscle index (PMI) was calculated based on psoas area using preoperative axial computed tomography images at the right L3 level divided by the square of the body height.

Results: Nineteen (76.0%) patients underwent partial penectomy, and 6 (24.0%) underwent total penectomy. The median (mean ± standard deviation) age was 69.3 (69.0 ± 10.1) years old. Regarding the site of penile cancer, 17 (76.0%) cases were in the glans, 6 (24.0%) were in the foreskin, and 2 (8.0%) were in the shaft. Lymph node metastasis were seen in 6 cases (24.0%), and distant metastasis was seen in 1 case (4.0%). The lower PMI group (< 320.0) showed a significantly poorer progression-free survival than the higher PMI group (≥ 320.0) (*p* = 0.030), although no significant difference in the overall survival was noted (*p* = 0.076).

Conclusions: Sarcopenia might be a useful prognostic factor in penile cancer patients.

## INTRODUCTION

Sarcopenia is defined as a low muscle volume due to aging [1]. It was initially investigated as a nutritional condition but recently has been studied as a risk factor predicting a longer hospitalization and increased risk of postoperative complications. We previously reported the importance of sarcopenia as a risk factor for a poor survival in bladder and renal cancer [2, 3].

Penile cancer is a rare entity, accounting for 0.5% of all cancer in men [4]. While localized penile cancer tends to have a relatively favorable outcome, lymph node metastatic cases show a poor prognosis. However, even cases of lymph node metastasis can show a favorable outcome with complete resection of the metastatic lymph node [5].

One previous study reported that sarcopenic penile cancer patients who underwent inguinal lymph node dissection showed a higher incidence of postoperative complications than those without sarcopenia [6]. We therefore examined the importance of sarcopenia as a new prognostic marker in penile cancer.

## RESULTS

A total of 25 penile cancer patients were included in this study, including 19 (76.0%) who underwent partial penectomy and 6 (24.0%) who underwent total penectomy. The median (mean ± standard deviation) age was 69.3 (69.0 ± 10.1) years old. The mean (range) observation period was 48.2 (2.3 to 118.5) months. Regarding the site of penile cancer, 17 (76.0%) cases were in the glans, 6 (24.0%) were in the foreskin, and 2 (8.0%) were in the shaft. Nine of 25 (36.0%) cases underwent lymphadenectomy and one case (4.0%) underwent biopsy for lymph node. Lymph node metastasis was seen in 6 cases (24.0%), and distant metastasis was seen in 1 case (4.0%). In these 6 cases, 5 of 6 (83.3%) cases underwent systemic chemotherapy and 1 of 6 (16.7%) case underwent radiotherapy for lymph node. One case showed distant metastasis and he received systemic chemotherapy. Other information, including the tumor grade, pathological T stage, Broders’ grade, Y-K grade, and clinical stage, is shown in Table 1.

**Table 1:** Psoas muscle index and parameters

Variables	Number (%) or Median (mean ± SD)			*p* value
	All	PMI>320	PMI<320	
Number of Pts.	25 (100%)	17 (68%)	8 (32%)	
Age	69.3 (69.0 ± 10.1)	68.1 (69.0 ± 10.4)	71.9 (74.0 ± 9.6)	0.390
Grade				
well	19 (76%)	13 (52%)	6 (24%)	0.722
moderately	5 (20%)	3 (12%)	2 (8%)	
poor	0 (0%)	0 (0%)	0 (0%)	
unknown	1 (4%)	1 (4%)	0 (0%)	
Tumor position				
G	17 (68%)	11 (44%)	6 (24%)	0.597
F	6 (24%)	4 (16%)	2 (8%)	
S	2 (8%)	2 (8%)	0 (0%)	
unknown	0 (0%)	0 (0%)	0 (0%)	
Broders grade				
1	9 (36%)	7 (28%)	2 (8%)	0.140
2	8 (32%)	7 (28%)	1 (4%)	
3	7 (28%)	3 (12%)	4 (16%)	
4	0 (0%)	0 (0%)	0 (0%)	
unknown	1 (4%)	0 (0%)	1 (4%)	
Y-K grade				
1	1 (4%)	1 (4%)	0 (0%)	0.722
2	9 (36%)	5 (20%)	4 (16%)	
3	6 (24%)	5 (20%)	2 (8%)	
4	8 (32%)	6 (24%)	2 (8%)	
unknown	1 (4%)		0 (0%)	
T stage				
1	14	11 (44%)	3 (12%)	0.638
2	7 (28%)	4 (16%)	3 (12%)	
3	2 (8%)	1 (4%)	1 (4%)	
4	2 (8%)	1 (4%)	1 (4%)	
Lymphnode metastasis	6 (24%)	3 (12%)	3 (12%)	0.278
Distant metastasis	1 (4%)	0 (0%)	1 (4%)	0.137
Stage				
1	11 (44%)	9 (36%)	2 (8%)	0.341
2	8 (32%)	5 (20%)	3 (12%)	
3	1 (4%)	0 (0%)	1 (4%)	
4	5 (20%)	3 (12%)	2 (8%)	

**Figure 1:**
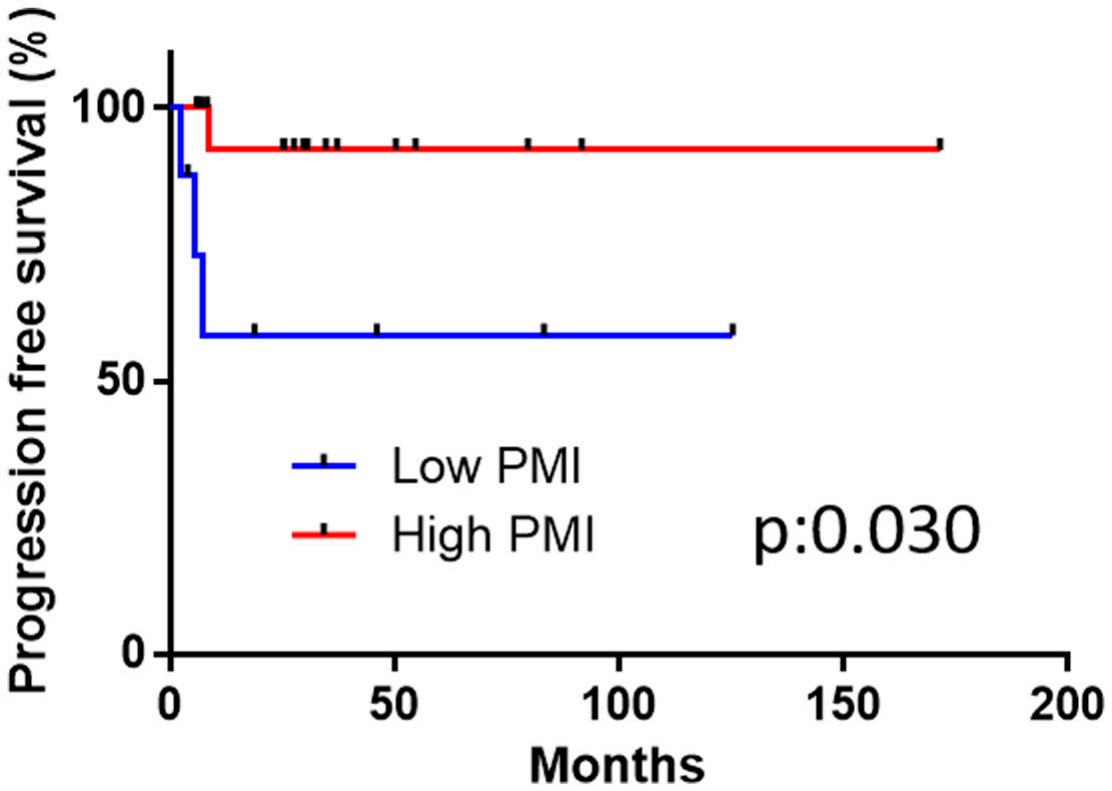
The disease-specific survival in the high and low psoas muscle index (PMI) groups.

**Figure 2:**
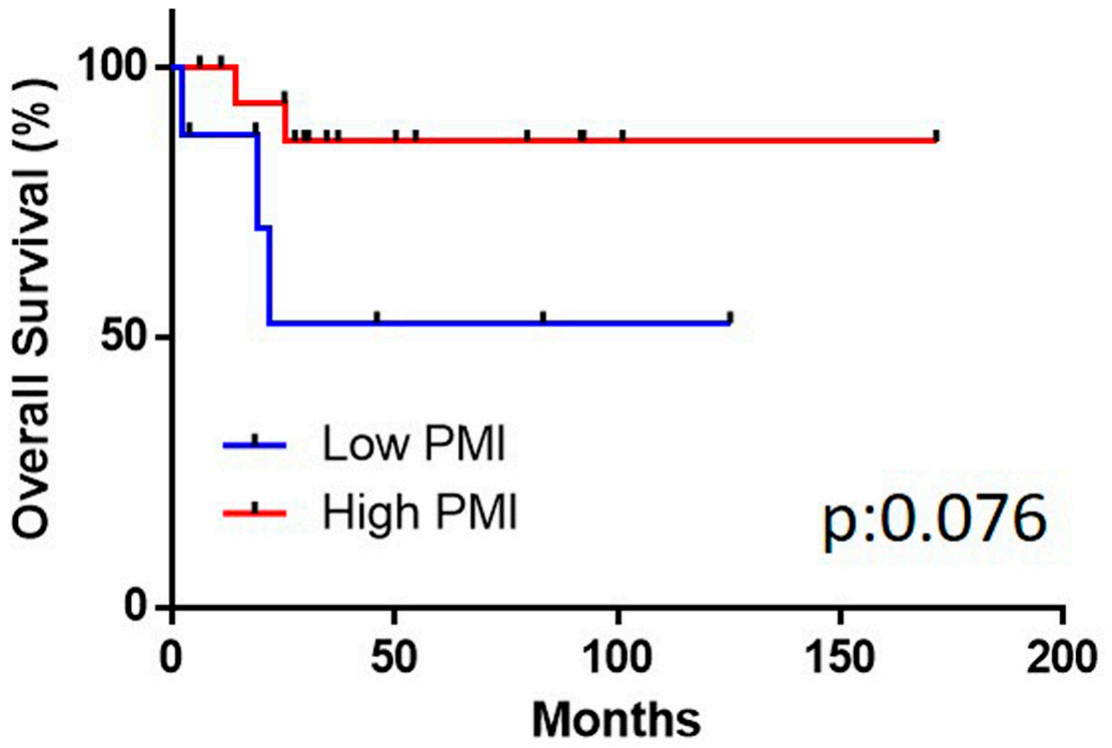
The overall survival in the high and low psoas muscle index (PMI) groups.

The ROC showed the candidate cut-off point of the PMI to be 320.0 (area under the ROC [AUC]: 0.6190) (Supplementary Figure 1). The lower PMI group (< 320.0) showed a significantly poorer PFS than the higher PMI group (≥ 320.0) (p:0.030). The median PFS in both the low and high PMI was not reached (Figure 1). The lower PMI group showed a poorer OS than the higher PMI group, although not to a significant degree (*p* = 0.076) (Figure 2). The median OS in both groups was also not reached. In higher PMI group, 3 of 17 (17.6%) cases received systemic chemotherapy and 1 of 17 (5.9%) case received radiotherapy. In lower PMI group, 3 of 8 (37.5%) cases received systemic chemotherapy and none of the patients received radiotherapy.

## DISCUSSION

Sarcopenia is defined as a decrease in the skeletal muscle mass and moving function [7]. It can be caused by multiple factors, including an advanced age, muscle loss, poor nutrition, inflammatory disease, and malignant tumors. In addition, female gender, liver dysfunction, and a low albumin level are risk factors for sarcopenia [8]. Sarcopenia lowers the performance status and increases the prevalence of neurologic disease, chronic lung diseases, and virus infection [9, 10]. Recent studies have revealed that a low PMI was associated with a poorer prognosis than a high PMI, including for cases of gastrointestinal, genitourinary, and gynecological cancers [8, 11–14]. However, no report has described the correlation between sarcopenia and penile cancer.

The relationship between sarcopenia and the PMI has been reported [15]. Sarcopenia has been defined using dual energy X-ray absorptiometry (DEXA), a bioelectrical impedance analysis (BIA), and CT [16–18]. We used CT in the present study because it was an easy way to investigate the PM volume at the time of the diagnosis retrospectively. The PMI is calculated by normalizing the psoas muscle area for the height in meters squared. Our previous report showed that the whole psoas volume was correlated with psoas area at the L3 level [2].

The present study revealed that a low PMI was associated with a poorer prognosis than a high PMI in penile cancer, findings that concurred with those of previous reports in other solid malignancies [8, 11–14]. However, our study did not describe the correlation between a lower PNI and other prognostic factors, including the pathological grade, T stage, Broders’ grade, and Y-K grade [19, 20]. Based on these findings, a lower PMI might be an independent risk factor for a poorer prognosis.

Several limitations associated with the present study warrant mention. First, this study was conducted with a relatively small sample size of 25 cases. Penile cancer is a relatively rare disease, occurring in 1.8 per 100,000 [21]. Thus, it would be difficult to investigate a large cohort. Second, we were unable to reveal the mechanism underlying the relationship between a low PMI and a poor prognosis in penile cancer. A low PMI has been thought to reflect anemia, a low BMI, inadequate nutrition, and the effects of other diseases [22]. Thought this findings, the molecular mechanism has yet to be clarified. Third, though significant differences were observed in PFS, there were no significant differences in OS. We speculated that due to the low incidence of penile cancer, not all patients were same background. And also this study included small number of patients, thus no significant difference was observed.

In conclusion, a low psoas muscle volume is associated with a poor prognosis in penile cancer.

## MATERIALS AND METHODS

A total of 25 penile cancer patients received penilectomy in Yokohama City University Medical Center (Yokohama, JAPAN) and Yokohama City University Hospital (Yokohama, JAPN) from 2000 to 2010 were enrolled in this study. The institutional review board of Yokohama City University approved this study (No. D1209028).

The psoas muscle index (PMI) was calculated based on preoperative axial computed tomography (CT) images at the L3 level using the following formula: [right psoas muscle area (mm^2^)]/[body height (m)]^2^. We compared the progression-free survival (PFS) and overall survival (OS) of the higher and lower PMI groups.

### Statistical analyses

The patients’ characteristics and preoperative factors were analyzed by Mann-Whitney U and chi-square tests. A receiver operator characteristic curve (ROC) was analyzed to determine the cut-off points for the PFS and OS. The Kaplan-Meier product limit estimator was used to estimate the PFS and overall survival (OS). The survival duration was defined as the time between penilectomy and death. The log-rank test was performed for comparisons. A *P* value of < 0.05 was considered to be statistically significant.

## SUPPLEMENTARY MATERIALS


