# Supplementary material for: A low psoas muscle volume is associated with a poor prognosis in penile cancer
Source: Oncotarget. 2020 Sep 22;11(38):3526–30. doi: 10.18632/oncotarget.27719 (PMC7517962; doi:10.18632/oncotarget.27719)
Supplement: Supplementary file 1 [file oncotarget-11-3526-s001.pdf]

A low psoas muscle volume is associated with a poor prognosis in penile cancer

SUPPLEMENTARY MATERIALS

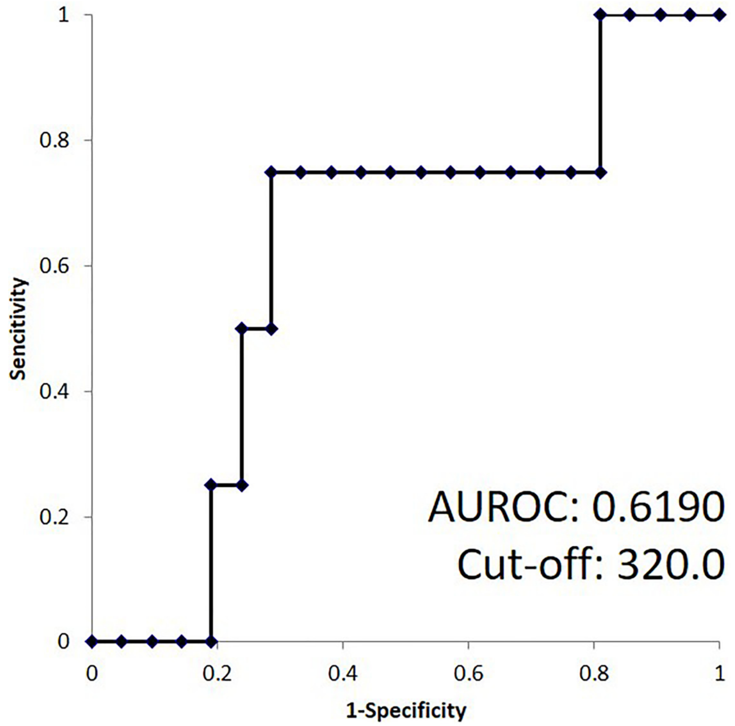

Supplementary Figure 1: Receiver operator characteristic curve.
